# An Ensemble Model for Consumer Emotion Prediction Using EEG Signals for Neuromarketing Applications

**DOI:** 10.3390/s22249744

**Published:** 2022-12-12

**Authors:** Syed Mohsin Ali Shah, Syed Muhammad Usman, Shehzad Khalid, Ikram Ur Rehman, Aamir Anwar, Saddam Hussain, Syed Sajid Ullah, Hela Elmannai, Abeer D. Algarni, Waleed Manzoor

**Affiliations:** 1 Department of Computer Science, Shaheed Zulfikar Ali Bhutto Institute of Science and Technology, Islamabad 44000, Pakistan; 2 Department of Creative Technologies, Faculty of Computing and AI, Air University, Islamabad 44000, Pakistan; 3 Department of Computer Engineering, Bahria University, Islamabad 44000, Pakistan; 4 School of Computing and Engineering, The University of West London, London W5 5RF, UK; 5 School of Digital Science, Universiti Brunei Darussalam, Jalan Tungku Link, Gadong BE1410, Brunei; 6 Department of Information and Communication Technology, University of Agder (UiA), N-4898 Grimstad, Norway; 7 Department of Information Technology, College of Computer and Information Sciences, Princess Nourah bint Abdulrahman University, P.O. Box 84428, Riyadh 11671, Saudi Arabia

**Keywords:** neuromarketing, EEG, SMOTE, LSTM, DWT, PSD

## Abstract

Traditional advertising techniques seek to govern the consumer’s opinion toward a product, which may not reflect their actual behavior at the time of purchase. It is probable that advertisers misjudge consumer behavior because predicted opinions do not always correspond to consumers’ actual purchase behaviors. Neuromarketing is the new paradigm of understanding customer buyer behavior and decision making, as well as the prediction of their gestures for product utilization through an unconscious process. Existing methods do not focus on effective preprocessing and classification techniques of electroencephalogram (EEG) signals, so in this study, an effective method for preprocessing and classification of EEG signals is proposed. The proposed method involves effective preprocessing of EEG signals by removing noise and a synthetic minority oversampling technique (SMOTE) to deal with the class imbalance problem. The dataset employed in this study is a publicly available neuromarketing dataset. Automated features were extracted by using a long short-term memory network (LSTM) and then concatenated with handcrafted features like power spectral density (PSD) and discrete wavelet transform (DWT) to create a complete feature set. The classification was done by using the proposed hybrid classifier that optimizes the weights of two machine learning classifiers and one deep learning classifier and classifies the data between like and dislike. The machine learning classifiers include the support vector machine (SVM), random forest (RF), and deep learning classifier (DNN). The proposed hybrid model outperforms other classifiers like RF, SVM, and DNN and achieves an accuracy of 96.89%. In the proposed method, accuracy, sensitivity, specificity, precision, and F1 score were computed to evaluate and compare the proposed method with recent state-of-the-art methods.

## 1. Introduction

It is a well-established practice to improve sales and awareness among consumers by marketing and promoting a variety of consumer products through an advertising campaign [1]. This can be done to increase sales. The understanding of the basic mechanisms that govern consumer shopping behaviors are the most essential topics that require further inquiry according to marketing professionals. In advertising and consumer behavior research, neuroscience can be utilized to improve the accuracy of existing marketing methods. Neuromarketing is a method that integrates physiological techniques and neuroscience to get insights into customer behavior in making accurate predictions of customers’ preferences during the process of making the choice [1]. Neuromarketing is an extremely new type of advertising that makes use of brain-imaging technology to investigate how peoples’ brains react to marketing stimuli. Electroencephalography (EEG) has been extensively used for decades to measure the activity of the brain neurons.

Consumer preference recognition by EEG signal is an inclusive and extensive topic of research. In order to understand why and how customers respond to stimuli, researchers use neuroimaging techniques to determine which areas of their brains are activated while making decision about the ecommerce product. By utilizing EEG signals, one can easily determine how the consumer truly makes his or her decision for buying the ecommerce product. Neuromarketing is a field of study that aims to better understand how consumers make their purchasing decisions. A company’s marketing strategy may be objectively improved based on what makes positive or negative impressions in consumers’ minds about their product [2]. Researchers [3,4] in the field of neuromarketing have emphasized the use of biometric data in marketing campaigns so that marketing companies and firms, by utilizing EEG signals, can have a better idea about the consumer’s brain activity while making purchase decisions. EEG signals in [5] were divided into different bands in the frequency domain. These bands have different frequency ranges and represent the following activities. The beta band (14–30 Hz) represents the occupied or busy brain. The alpha band (8–14 Hz) shows the calmness of the brain. The theta band (4–8 Hz) reflects the excitement. The delta band (1–4 Hz) reflects sleep, relaxation, and fatigue [1].

The term neuromarketing refers to the combination of two disciplines, namely neuroscience and marketing. The field primarily makes use of medical technologies in order to conduct studies into the responses of the brain to varying conditions. If a person takes an example of traditional advertising methods used by different companies, when a consumer buys an ecommerce product and expresses interest or loses interest in something, one can only obtain the consumer’s point of view about a certain item and has no knowledge of the activities taking place in the consumer’s subconscious at the time of the purchase. As a result, a person is unable to distinguish between the preferences of customers who like or dislike the product [6]; in this circumstance, EEG signals can be used to determine the customer preference about the product. Approximately 90% of data is reportedly processed subliminally in the human mind [7]. If we consider the fields of neuromarketing and consumer neuroscience, the conventional marketing research methods cannot obtain insight into the subconscious activities of customers. The information that can be acquired by the use of neuromarketing is also more accurate than the information that can be retrieved from traditional approaches. This is due to the fact that consumers’ decisions are influenced by their subconscious beliefs. Because traditional market research does not concentrate on the subconscious processes that occur in a customer’s brain while they are making a purchase decision, there is a disparity between the findings of traditional market research and the actual behavior of customers at the point of sale. This results in a gap between the two sets of data [8]. The main contributions of this research study are as follows.

- The proposed method has achieved significant improvement in results due to noise removal from EEG signals.
- The class imbalance problem has been resolved by the help of the synthetic minority oversampling technique (SMOTE).
- The proposed method has been able to recognize consumer choice in terms of like and dislike with accuracy 96.89%, sensitivity 95.89%, and specificity 96.21%.
- A new ensemble classifier has been proposed in this study that has never been used before in existing methods, and it helps to accurately classify EEG signals between the like and dislike classes.

The research paper is organized in different sections. A literature review presents necessary background of the problem and a detailed literature review about the state-of-the-art methods for customer preference recognition. The literature review is divided into three major components. Starting with the discussion about preprocessing techniques used by different studies, we follow up with a summary of various feature-extraction techniques. An overview of the various classification methods is presented, employed by different researchers. The Materials and Methods section consists of an introduction to the publicly available neuromarketing dataset. Signal acquisition is discussed in detail followed by the steps taken to prepare, structure, and store the data. The proposed methodology embodies a complete methodology of the proposed mechanism. We first provide an overview of the proposed system along with a flow diagram. The rest of the sections include proposed feature extraction, in which we discuss handcrafted feature-extraction methodologies and automated feature extraction by using LSTM. The classification section explains the techniques employed for classification of EEG signals like DNN as a deep learing classifier and SVM, DT as a machine learning classifier, and an ensemble model for classification of EEG signals into like and dislike.

Neuroscience methods have enhanced marketing strategies in the last century by allowing researchers to examine both conscious and unconscious influences on consumer behavior. Due to its low cost, EEG is one of the most commonly used neuroscientific tools in marketing studies [9]. In most of the cases, customers are not compelled to buy goods when conventional marketing methods (e.g., television ads and newspaper ads) are used. Marketing strategies such as television commercials, newspaper advertising, and brochures merely try to determine a person’s attitude toward a product; this attitude may or may not match the person’s real behavior when it comes time to make a purchase. The goal of this study is to determine the preferences of customers in terms of their likes and dislikes by analyzing the EEG signals that are generated by the customers’ brain activity.

Consumer buying behavior is the foundation of both traditional advertising research and neuromarketing studies [10]. In spite of the similar starting premise, the research methods used by the two methodologies differ significantly. These discrepancies are the result of varying research approaches in both fields. In conventional marketing research methods, we analyze the product which is already launched in the market, but in neuromarketing research we analyze the product from different aspects which have yet to be launched in the market. The consumer self-reports are very important in conventional marketing research, but in neuromarketing the consumer’s personal reviews about the product are not as important because we are gathering the brain activity of the consumer.

In neuromarketing research, the reactions of the consumers are not controlled, but in conventional marketing research the reactions of the consumer are controlled. In the conventional marketing research the participant has time to study the research questions before answering them, but in neuromarketing research the participant or consumer’s physiological reactions can be gathered immediately as he/she is presented with the research questions about the product. Mostly, people are reluctant to completely convey their opinions and preferences about a product when asked directly, so one does not know what actually is happening in the subconscious of consumers when employing conventional marketing methods. However, there are various neuroimaging tools that can easily access the consumer brain information while making decisions or expressing preferences for different products. In this way, brain-imaging techniques and tools can help marketers and advertisement agencies to improve the marketing campaigns before launching the product in the market and also during the in-market inspection of campaign’s success after the launch.

## 2. Literature Review

Most people are unwilling to express their whole thoughts and preferences about a product, so one can have no idea what is going on in the mind of a consumer when making purchase decisions. Neuroimaging tools make it possible to obtain information quickly and readily about a customer’s brain while they are evaluating various products and making purchase decisions. Consumer choice recognition basically involves three main steps. The first step is preprocessing in which unwanted noise will be removed from EEG signals, the second step is to extract the desired features and then comes to classify EEG signals in terms of likes and dislikes. In neuromarketing studies, consumers’ brain signals are recorded so that researchers can better understand how the human psyche chooses one item over another.

### 2.1. Preprocessing of EEG Signals

In any kind of machine learning application, data is usually in raw form and needs some sort of preprocessing before it is usable for feature extraction. For many decision-making sectors, the automatic analysis of diverse and multimodal data and the instantaneous extraction of information by using machine learning approaches have become major challenges [11,12,13,14].

In particular, a wide variety of artifacts, including eye blinks, muscular activity, and noise from electrical power lines, might emerge during EEG signal recording (see Gauba et al. [12]). Such artifacts could distort useful information in the signal; thus, it is necessary to delete them to get better results. For preprocessing, several techniques have been discussed here. Amna et al. [13] removed the noise from EEG signals by using independent component analysis (ICA). Abeer et al. [2] used bandpass filter for noise removal. Aldayel et al. [5] removed noise with a Savitzky–Golay filter.

Gupta et al. [15] found that using a notch filter operating at either 50 or 60 Hz significantly reduced the amount of electrical and environmental interference. The elimination of artifacts was accomplished by Yilmaz et al. [9]. Many researchers have turned to bandpass filtering with a variety of cutoff frequencies so that the quality of the signal can be improved before it is employed for prediction. Rakshit et al. [16] used Butterworth fourth-order bandpass filters with cutoff frequencies ranging from 0.5 to 60 Hz in their research. Preprocessing of EEG signals has been accomplished by using tenth-order elliptical bandpass and common average referencing spatial filters [17]. ICA and principal component analysis (PCA) are two other techniques for removing artifacts (see [18,19], respectively).

**Figure 1:**
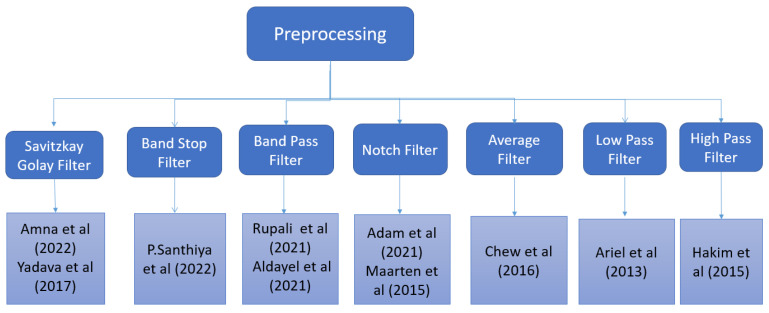
Preprocessing of EEG signals in literature.

Figure 1 shows us the preprocessing techniques employed for removing noise from raw EEG signals.

### 2.2. Feature Extraction of EEG Signals

Data that has been preprocessed typically consists of large quantities and has higher dimensions. Data presented in this way does not convey any information that is valuable and also provides redundant information. The term “feature set” refers to a subset of data that contains fewer dimensions, and additional processing is done on this feature set. The transformation of data into a feature set is known as feature extraction. When the EEG signals have been preprocessed, features are extracted for the classification between like and dislike states. EEG signals are decomposed by the Daubechies 4 wavelet decomposition in [5]. Abeer et al. [2] extracted features by using PCA. Aldayel et al. [5] splits the coefficients into five frequency bands. Reference [20] used Morlet wavelet transform by using Gaussian wave shapes. Reference [20] have employed the FFT for feature extractions, and STFT was used by Rakshit et al. [16].

**Figure 2:**
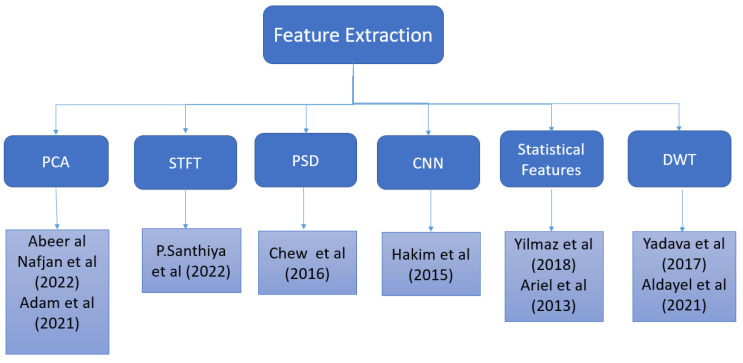
Feature extraction of EEG signals in the literature.

DFT was employed by [23] for feature extraction.The statistical mean was calculated by [21] for all electrode channels, whereas [18] only utilised it for the specific channels. In [21], the Welch method was used for feature extraction. According to Guo et al. [22], there are two ways to estimate rating. One of them is to take the average of the relative power measures among participants, and the other is to simply use the average relative power across participants. The power spectrum density has been extracted by numerous researchers like [19,23,24,25], and power spectral analysis has been used to obtain spectral moments. In [26], features were extracted by statistical analysis by employing the spectral centroid. Figure 2 shows us the techniques employed for extracting features from EEG signals.

### 2.3. Classification of EEG Signals

Following the completion of the process of feature extraction from EEG signals, the next step is to categorize the signals into the like and dislike states. One definition of classification is the process of developing a model that partitions the data into a number of distinct categories. The values of particular distinguishing characteristics are used to classify the data; samples that belong to the same class as other samples in terms of these characteristics’ values are classed together. By using a boosted tree classifier, Amna et al. [27] were able to reach an accuracy of 88.89% when classifying EEG signals. Abeer et al. [2] employed DNN for the classification of EEG signals.

Researchers [25,28] employed RNN for the EEG data classification model. Ref. [29] classified EEG with 92.40% accuracy into four categories of movements (foot, right/left hand, and rest) by using advanced visualization techniques and a convolutional neural network (CNN) [30,31]. In [32] researchers employed a CNN model for classification of EEG signals. Hasnain et al. [33] extracted features from EEG data by using a convolutional deep belief network. The parietal lobe is responsible for touch, taste, and bodily awareness. The prefrontal and frontal lobes have the most influence on neuromarketing [34]. Certain studies focused their efforts on certain brain regions [35], whereas others considered the entire brain [34,36,37,38,39,40].

**Figure 3:**
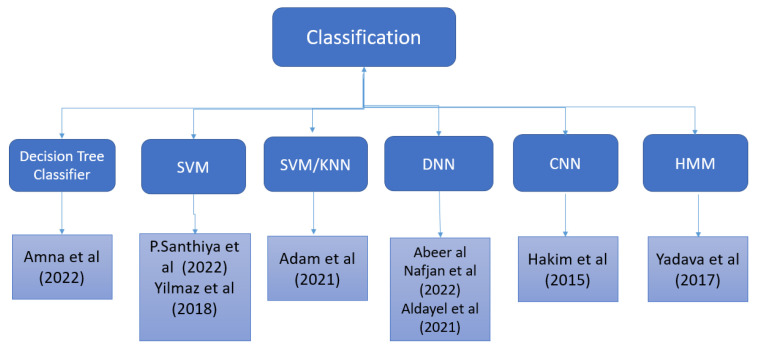
Classification of EEG signals in the literature.

Ambler et al. [34] discovered that advertisements had an effect on brain activity in diverse cortical areas. Researchers demonstrated the effect of visual stimuli on the activation of the left frontal lobe by using EEG in the study cited in the previous sentence [37]. Dmochowski et al. [41] analyzed EEG data from participants in commercial videos in order to identify regions of the brain that are consistently more (or less) active in response to stimuli. Braeutigam et al. [42] goes into additional depth on both predictable and unpredictable decisions, where predictability is determined by prior use of the product and the time gap between stimulation and decision making. The multiple brain regions that are related to pleasure and reward were investigated in [43], and the study provides a comprehensive explanation of these brain regions. Researchers used a mix of convolutional neural networks (CNNs) and long short-term memory (LSTM) in [44] to classify emotions based on EEG readings. Figure 3 provides a representation of the classification methods that researchers used in order to divide EEG data into preference categories of like and dislike.

Most of the research in neuromarketing and EEG is focused on how consumers feel about products, but here the emphasis is on the details of the product that cause the subject to make a particular choice (see Fernandez et al. [45]). Duan et al. [46] employed PNN and KNN for classification of EEG signals. Brain activation and oscillatory activity between the left and right occipital electrodes were studied by Kawaski et al. [47] to better understand the impact of color preference on the visual attention-related region of the brain. Rakshit et al. [16] employed logistic regression to discover the most distinct frequencies for consumer product choice. Frontal spectral activations of the brain have been explored by looking at the subjects’ preferences, as they record them (see Kawasaki et al. [48]). On a diabetic retinopathy dataset, a model composed of machine learning (ML) algorithms such as random forest (RF) classifier, decision tree classifier, adaboost classifier, K-nearest neighbor classifier, and logistic regression classifier is tested by Reddy et al. [49]. Aldayel et al. [5] features are classified into like and dislike by using SVM and RF and obtained an accuracy of 68.33%. Morin et al. [50] conducted research and used the Welch method for classification of EEG signals. Yadava et al. [1] used the HMM for classification of EEG signals in terms of likes and dislikes. Aldayel et al. [5] used the DNN for the classification of EEG signals. In [51], researchers used SVM for the purpose of classification. Luis et al. [52] also used SVM for classification. In Hammou et al. [7], the researchers used RF for classification.

Classification is the ultimate and also the most cardinal step in consumer choice recognition systems [53], as it is the classifier performance that is used to calculate the sensitivity and specificity. Artificial neural networks, deep neural networks, linear discriminant analysis, K-nearest neighbors, RF, and decision trees were mostly used by researchers in existing methods. A comparison of the most recent state-of-the-art consumer choice recognition approach demonstrates that preprocessing of EEG signals is essential for the classification of EEG signals with high sensitivity and specificity. In the feature-extraction process, both handcrafted and automated features can be extracted; nevertheless, it has been noted that automated features outperform handcrafted features.

A combination of both of these characteristics can be advantageous, but it is not currently utilized by researchers in their techniques employed in existing choice recognition systems. Feature selection also minimizes the influence of the curse of dimensionality, which is lacking in current approaches. Furthermore, there is a tradeoff between sensitivity and specificity. Multivariate features can be retrieved, and classification can be performed by using DNN, SVM, and RF as these classifiers provide improved performance if preprocessing and extraction of features have been performed effectively.

Analysis of the existing state-of-the-art methods has shown that choice recognition cannot be predicted with higher sensitivity without efficient preprocessing, a complete set of features, and effective classification. Existing approaches in all three processes are hindered by numerous research gaps. In preprocessing, many researchers do not use a set of methods to boost the SNR of EEG signals. No technique for EEG signals has offered a solution to reduce the impact of the class imbalance problem on consumer choice recognition. Existing approaches lack a comprehensive feature set, which must be created by combining both handcrafted and automated features, and classification has also been kept simple. Table 1 shows the recent state-of-the-art methodologies for consumer emotion prediction by using EEG signals.

## 3. Materials and Methods

### Dataset Explanation

The dataset was recorded by [1] by using an Emotiv EPOC+ device and consists of EEG recordings from 25 subjects for 42 different products. Table 2 shows a summary of the dataset. Electrodes placed on the scalp provide different channels of brain signals. A total of 14 electrodes were used for the acquisition of the EEG signals. A sampling frequency of 2048 Hz is used internally in an EPOC and then the data is downsampled to 128 Hz in order to reduce data and speed up computation. Table 2 depicts the details about the neuromarketing dataset gathered by Yadava et al. [1].

## 4. Overview of Proposed Methodology

In the proposed method, preprocessing involves artifact removal and noise removal using the Savitzky–Golay filter. This filter is solely responsible for smoothing EEG signals. Smoothing data is a method of removing noise from a set of data. It enables the creation of a pattern that stands out from the ambient noise. A band stop filter has been applied on this frequency domain data to remove noise. The SMOTE algorithm is also employed to deal with the class imbalance problem.

**Figure 4:**
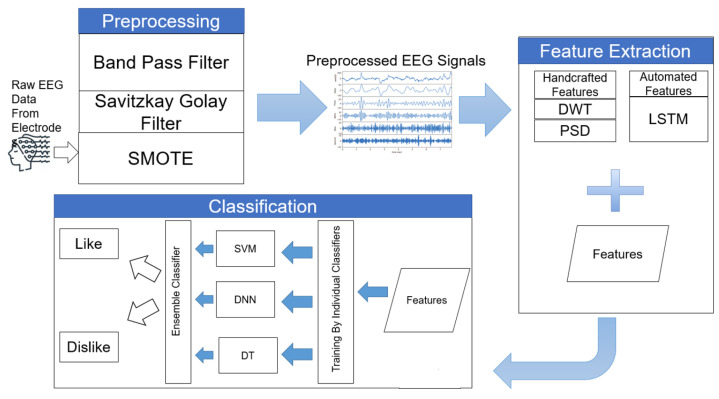
Flow diagram of the proposed consumer emotion prediction.

SMOTE makes use of the vector interpolation approach in order to produce synthetic samples of the minority class when working with high-dimensional data. Following the completion of the preprocessing, the features are extracted through the use of the power spectral density (PSD), also known as the Welch method, and the discrete wavelet transform as examples of handcrafted features, and long short-term memory (LSTM)-based features as examples of automated features. In signal processing applications, LSTM is extensively applied to extracted automated features. After extracting features from denoised EEG signals, the training data was 70% and testing data was 30%. Different ML classifiers were employed, including decision tree (DT), SVM, and deep learning classifiers (DNNs) for classification between the two classes. However, it is observed that the ensemble classifier gives better classification results in terms of increased sensitivity and specificity. Figure 4 shows us the proposed ensemble model for consumer emotion prediction by using EEG signals.

### Proposed Preprocessing of EEG Signals

EEG recordings are easily influenced by external noise. It is difficult to pinpoint the features in EEG signals due to noise. There are a variety of known solutions to the noise problem. Smoothing data is a method of removing noise from the EEG signals to increase the signal-to-noise ratio. It enables the creation of a pattern that stands out from the ambient noise. Noise was removed from EEG signals by using the Savitzky–Golay filter that smoothes the signal and removes the noise. FFT and the Savitzky–Golay filter were employed to reduce noise and remove artefacts. A Savitzky–Golay filter is a digital filter that can be used to a range of digital data points to smooth them out to increase their precision without affecting the signal. In the dataset, there are two classes, like and dislike. Another problem is the like-to-dislike samples ratio, which indicates that there are very few samples of the like class available in the dataset in comparison to the samples available for the dislike class. This leads to a class imbalance problem, which in turn hinders the classification’s performance.

As part of the proposed method, synthetic data for the like class has been generated in order to lessen the impact of the class imbalance between the like and dislike classes. To overcome the problem of class imbalance, SMOTE was employed to generate the samples for the like class. In the SMOTE technique, the oversampling technique was employed, in which the data of the minority class was duplicated from the majority class population. SMOTE works by using a *k*-nearest neighbor algorithm to make synthetic data samples for the minority class. The samples are exactly the same as the original samples. SMOTE works on selecting instances in the feature space that are close to each other, drawing a line between them, and then drawing a new sample along that line. First, an example from the minority group is selected randomly. Then, *k* of the nearest neighbors for that case (usually k=5) are discovered. Synthetic samples are constructed at random points between the two samples in feature space, based on a random selection of a neighbour.

After applying the SMOTE technique and removal of noise to increase the SNR, the frequency was resampled to a 128 Hz channel. In contrast to previous findings, preference states seem to generate low-frequency EEG signal ranges primarily. Thus, the useful bandwidth of the EEG signal data for choice detection is between 4 and 45 Hz. A bandpass filter with a bandpass of 4.0 to 45.0 Hz was applied. Savitzky–Golay filters accept a variety of input parameters, including *X*, order, and frame length. We employed a Savitzky–Golay filter with an order of 11 and a frame length of value 2. To eliminate noise from all 1050 files, a Savitzky–Golay filter was employed. We have

(1) $$Q_{j}=\sum_{{}_{i=-\frac{m-1}{2}}}^{{}_{\frac{m-1}{2}}}c_{i}S_{j+1},\frac{m+1}{2}\leq j\leq n-\frac{m-1}{2}\left(3.1\right),$$

where *m* is the number of frames, $c_{i}$ is the number of convolution coefficients, and *Q* is the smoothed signal. Polynomial values are calculated with the frame span *m*, which is used to find the values of $c_{i}$ with this method.

## 5. Proposed Feature Extraction of EEG Signals

Feature extraction is the process of getting lower-dimension, useful, and nonredundant information from the data. This reduced set of information is known as the feature vector. Automated feature extraction techniques and handcrafted feature-extraction methods were employed for getting better results. Feature extraction is a procedure that enhances the complexity of raw EEG signals, and by the help of feature extraction, one easily gather required information from the EEG signal. Many time-frequency domain feature-extraction approaches are available. Wavelet transform (WT) for EEG signals is now the most common and useful option. The proposed method calculates features in both domains like the time domain and frequency domain.

### 5.1. Handcrafted Features

The preprocessing of EEG signals is followed by feature extraction. Discrete wavelet transform and power spectral density are handcrafted features extracted from EEG signals. DWT encodes the signal in the time-frequency domain and are usually applied in biomedical signal processing. When it comes to decomposition, the DWT method uses a multistage approach to break down an input signal into smaller waves.

Wavelet transform methods are of two types, namely CWT and DWT [60]. DWT stands for discrete wavelet transform, and it is a wavelet transform that samples wavelets by using scaling and translation parameters. The wavelet vector decomposes the signals into orthogonal components (see Nilashi et al. [60]). The DWT technique derives a collection of features, which includes details (D2-D5) and (A5). The signals are decomposed into wavelet coefficient vectors, which are then analyzed. Both time and frequency domains are considered in this technique. The following two equations describe the sequential filtering of the initial signal, which begins with low-pass filtering *g* and ends with high-pass filtering *h*. The wavelet function can be seen as in following equations,

(2) $$\int_{+\infty }^{-\varphi \infty }\psi \left(t\right)dt=0$$

(3) $$\varphi_{m,n}\left(t\right)=a_{0}^{\frac{-m}{2}}\varphi \left(a_{0}^{-m}t-nb_{0}\right),$$

where *a* and *b* are scaling and translation parameters that can have discrete values. *m* is frequency and *n* is time belonging to *Z*. *A* and *D* are shown in the scaling function (4), and (5) denotes the wavelet function. We have

(4) $$\phi_{j,k}\left(n\right)=2^{j/2}h\left(2^{j}n-k\right)$$

(5) $$w_{j,k}\left(n\right)=2^{j/2}g\left(2^{j}n-k\right).$$

($\phi_{k}$), k(n) denotes the scaling function that belongs to (L), and ($\omega_{j}$), k(n) denotes the wavelet function that is related to (H); the signal’s length is denoted by *M* Here, *n* is the discrete variable that lies between the values of 0 and *M-1* and here we have *J* = (log2) (M) and the values of *k* and *j* are between *0-J-1*. Equations (6) and (7) are used to calculate the values of Ai and Di [27],

(6) $$A_{i}=\frac{1}{\sqrt{M}}\sum_{n}x\left(n\right)\times \phi_{j,k}\left(n\right)$$

(7) $$D_{i}=\frac{1}{\sqrt{M}}\sum_{n}x\left(n\right)\times \omega_{j,k}\left(n\right).$$

**Figure 5:**
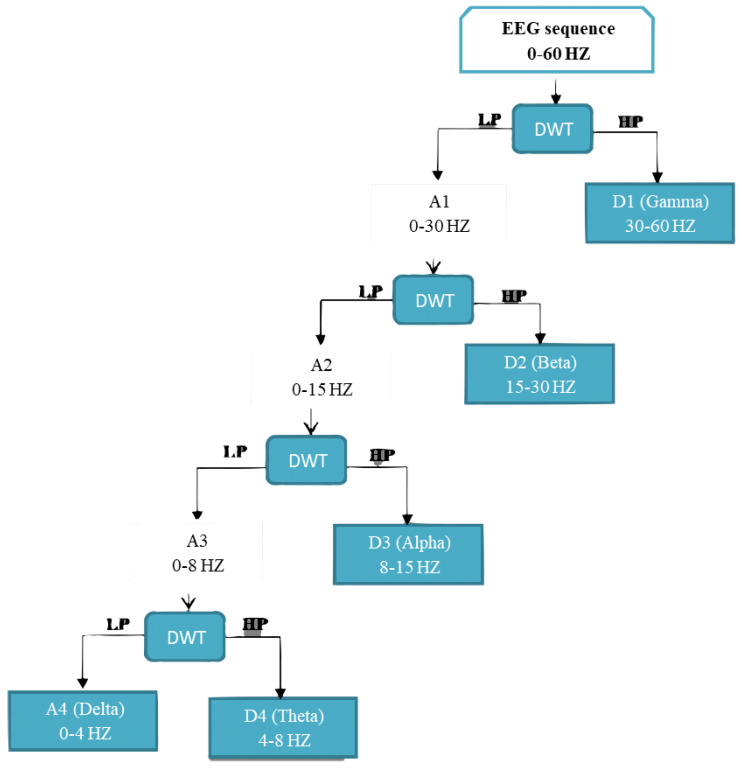
Decomposition of EEG signal into four levels by using DWT.

Figure 5 shows the four-level decomposition of EEG signals. A second feature-extraction method utilised is PSD. Fourier analysis demonstrates that every physical signal may be reconstructed into a spectrum of frequencies spread across a continuous range. The signal’s frequency content, including noise, is called its spectrum. When a signal’s energy is focused in a certain time span, the energy spectral density can be computed. The PSD can be defined as the energy distribution per unit time in a signal because the total energy of a signal throughout all time is limitless.

The PSD indicates the strength of a signal by its frequency. PSD is a technique that is widely used in neuromarketing research is feature extraction by frequency domain analysis [61]. For determining power spectral density of EEG signals, MATLAB was used.

### 5.2. Automated Feature Extraction by Using LSTM

Predictive problems using time series data are notoriously challenging to implement due to their inherent complexity. Time series predictive modeling adds more complexity than traditional regression predictive modeling because it includes a sequence dependence among the input variables. One of the most effective types of neural networks, recurrent neural networks, are able to take sequence dependency into account. Recurrent neural networks like the LSTM network are popular in deep learning because they allow for the successful training of extremely massive architectures. The inability to selectively remember essential information or values for a longer period of time causes feed-forward neural networks and non-LSTM recurrent neural networks to be less effective at sequence prediction.

**Figure 6:**
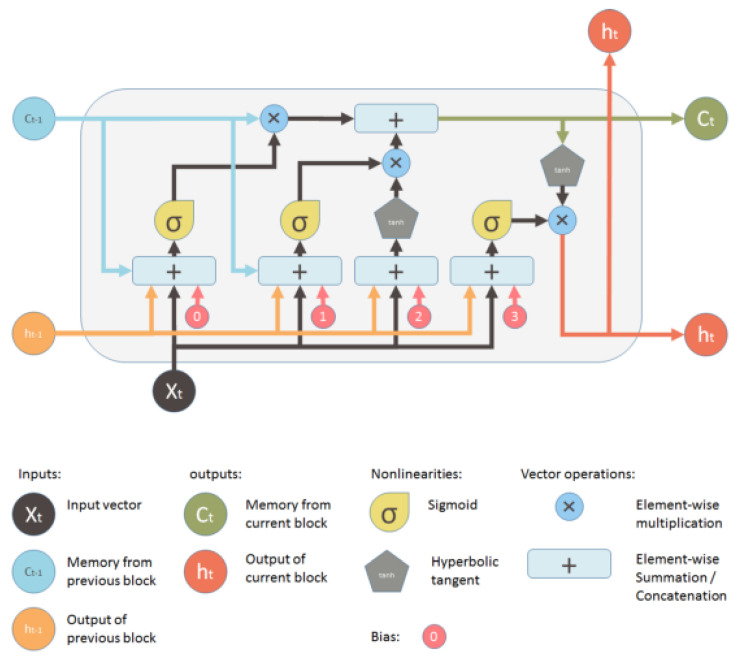
Architecture of LSTM model [36].

In 1997, Sepp Hochreiter and Jürgen Schmidhuber published the recurrent neural network (RNN) architecture known as LSTM [62]. When it comes to learning from experience, conducting analysis, and making predictions about time series data, LSTM networks excel in comparison to traditional RNNs. Several iterative enhancements have been made to LSTM designs over the years. The LSTM architecture relies heavily on the idea of gated cells. The architecture of the cell allows LSTM to handle long-term dependence by regulating the influx and egress of data. There is a cell state and three gates in an LSTM cell. Figure 6 shows the architecture of the LSTM model employed for automated feature extraction.The purpose of the sigmoid function used by each gate is to either add or subtract data from the current state of the cell.

A memory cell is composed of four primary elements: an input gate, a neuron with a self-recurrent connection, a forget gate, and an output gate. These elements work together to form the cell. A memory cell’s state is guaranteed to be stable from one time step to the next thanks to the self-recurrent link’s weight of 1. The gates control how the memory cell communicates with its surroundings. The state of the memory cell can be altered by external signals, which can be allowed or blocked by the input gate. In contrast, the state of the memory cell can either impact other neurons or be blocked by the output gate. Both of these outcomes are possible. The forget gate has the ability to alter the self-recurrent link that is present in the memory cell, causing the cell to either remember or forget its former state.

### 5.3. Significance of Using LSTM for Automated Features

In recent years, deep learning and machine learning technologies have gained prominence, with considerable impacts seen in real-world applications such as image/speech recognition, NLP, classification, prediction, and a wide variety of other applications [63]. The development of artificial neural networks has made these kinds of things conceivable in recent years. RNNs, which are one sort of advanced artificial network, have a great deal of flexibility as a result of their capacity to carry out operations on sequences. When it comes to understanding data patterns that change over the course of time, an RNN is the best option. It is widely acknowledged in the field of data science that the prediction and categorization of sequences is one of the most difficult problems to solve. In time series data, these challenges can range from estimating sales to seeing trends in stock market data, from comprehending movie plots to recognizing voice tones, from translating languages to predicting a typist’s next word on the keypad of an iPhone, etc. In light of current developments in data science, it has come to light that LSTM networks are the most efficient solution to the vast majority of these sequence prediction or classification issues.

The fully connected layer in an LSTM is used to extract robust and relevant features, whereas the Softmax layer in LSTM is used to extract predicted labels in output. Due to the RNN’s recurrent structures, LSTM has a low computational complexity when using gradient-based learning techniques to train a neural network. Vanishing gradient is a typical problem that hinders the network’s capacity to learn and perform. Although RNNs provide a great deal of resilience, their limited memory means they are vulnerable to vanishing gradients. In order to fix this, a more suitable structure, such as an LSTM is required. The latter is a complex design that overcomes vanishing gradient problems; it is a version of an RNN. Remembering the previous inputs is essential because the output is based on those inputs. When more parameters are introduced, the standard RNN’s inability to look back more than a few time steps hinders its performance. An LSTM may selectively forget and remember data/patterns for very long period of time. The ability of an LSTM to detect and prioritize which input and information should be kept within the network sets it apart from more basic RNNs and feedforward neural networks.

## 6. Proposed Classification

After the successful completion of the training process of automated features by LSTM and handcrafted features by using DWT and PSD, the data was passed to our hybrid classifier. The hybrid classifier combines the weights of SVM, DT, and the deep learning classifier DNN. It gives the findings of each individual classifier a weight and then utilizes this weight-probabilistic ensemble for further processing. The data that was utilized for training is 70% and testing was done on 30% of the data comprising two labels, namely like and dislike. Figure 4 depicts the proposed ensemble framework for the hybrid classifier. The proposed methodology consists of classifiers, namely SVM, DT, and DNN. A genetic algorithm is used to optimise the weights in this weight vector. The weight-modelling process is divided into two stages. The separation of incorrect samples from the rest of the samples in the training dataset is done in the first phase. When individual classifiers classify samples, they classify them into distinct classes, resulting in incorrect samples. These samples were employed alone for optimisation purposes. As we know that processing only the confused samples requires less time, the weights for the confused samples determined in the previous step are optimised in the second phase and optimization of weights was done by genetic algorithm. The detailed working of individual classifiers are as follows.

Decision tree refers to a method of supervised learning that is nonparametric and can be used for both classification and regression. The characteristic that results in the greatest increase of information is chosen to serve as the root node for the [64] structure. Information gain is defined as the anticipated decrease in entropy that is brought about as a result of dividing the samples up according to this characteristic. The following equation can be used to determine the entropy of a system. We have

(8) $$Entropy=-\sum P_{i}log_{2}\left(P_{i}\right),$$

where $P_{i}$ is the ratio of elements in of each label in a set.

The SVM refers to a group of supervised learning algorithms that can be used for classification, regression, and the identification of outliers. SVM is useful in high-dimensional spaces, and their usefulness does not diminish even in circumstances in which the number of dimensions exceeds the number of samples. However, the fundamental concepts that underpin the SVM algorithm can be described in a way that does not involve the use of equations. We have

(9) $$k\left(x,y\right)=exp(-||x-y|{|}^{2}/\sigma^{2})$$

(10) $$k\left(x,y\right)={\left(ax\cdot y+b\right)}^{n}$$

(11) (x,y)=exp(−a||x−y||+b)

(12) $$k\left(x,y\right)={\left(a||x-y||+b\right)}^{1/2}$$

(13) $$k\left(x,y\right)={\left(a||x-y||+b\right)}^{-1/2}.$$

These equations are for the different kernels for SVM. In the SVM, separating hyperplanes are selected based on their margins, which are defined as the distances from the separating hyperplane to the nearest expression vector. By using this hyperplane, the SVM is better and can be able to anticipate the right categorization of samples that have not been seen before.

Once the removal of noise from the data is complete, we split the data by using the train–test split into 70% for training and 30% of the data for testing purposes. We do this before feeding this data to the deep learning model for extraction of features. The number of epochs, also called hyperparameters, is 300. Before starting the training process, the values of hyperparameters must be defined, these values express the model’s layer size and decides how the model is being trained.

The hyperparameters that are defined for the DNN classifier are batch size = 100 and epochs = 300. These defined hyperparameter values are optimal, but in some cases these values may not be optimal. Hence, along with the tuning of these sets of hyperparameter values, we have obtained optimal results; this procedure is called hyperparameter tuning. The loss was found with an optimizer which reduces the loss function. Adam is an optimization algorithm for stochastic optimization. For binary class classification, the binary class entropy was employed. An algorithm called binary cross-entropy evaluates each prediction to the actual class output, which can be either 0 or 1. It then creates a score that penalizes the probability based on the difference between the expected and actual values. Increasing cross-entropy loss occurs when the anticipated probability diverges from the actual label. A comprehensive feature vector can be obtained by first extracting the automated features from EEG signals. DNNs are models that are made up of layers of ”neurons” that are connected together and in which each layer performs a linear change to the input data. A nonlinear cost function is used to handle the transformation results of each layer after they have been transformed in each layer. By minimizing a cost function that describes the transformation, one can establish how the parameters of such transformations should be set. The applications of deep learning covered an extremely broad spectrum, including areas like as speech recognition, image recognition, and the processing of natural languages. It has been demonstrated that DL is an excellent method for analyzing EEG signals. To detect the consumer preference in terms of likes and dislikes, a model is built based on handcrafted and automated features extracted by DWT, PSD, and LSTM.

The DNN model is a feed-forward neural network with five hidden layers which are fully connected. The input layer has 512 input units, whereas each subsequent hidden layer had 20% of the previous layer units. The activation that was employed is a rectified linear unit (ReLu). The cross-entropy function or cross-functions was employed to calculate the output was SoftMax. The number of target preferences (2) was correlated to the dimension(s) of the output layer. As we are detecting two states from EEG signals, like and dislike, we have two units in our output layer. We have employed the Adam gradient descent on the DNN classifier to train it with the following characteristics: three different objective loss functions (binary cross-entropy, categorical cross-entropy, and hinge cross-entropy). The dropout rate for the input layer and hidden layer was 0.3%. We have also used the early stopping criteria to overcome the overfitting. The test set had around 30% of the samples in the dataset; therefore we tested our classifier on it.

### Ensemble Classification by Using the Genetic Algorithm

**Figure 7:**
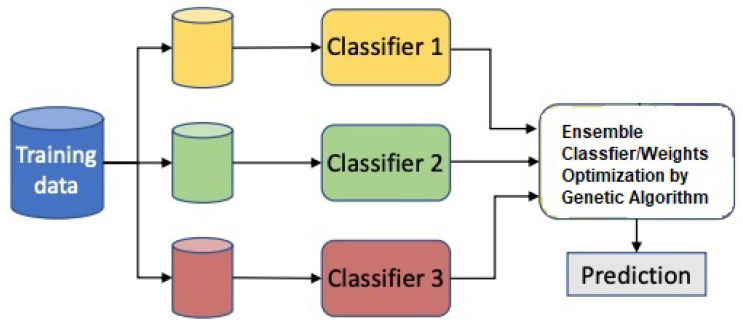
Block diagram of ensemble classifier.

After the successful feature extraction, two types to features were gathered. The first one is handcrafted features extracted by DWT and PSD, and the second are automated features using LSTM. The proposed methodology consists of classifiers, namely SVM, DT, and DNN. Weight optimization was carried out by using the genetic algorithm. It gives the findings of each individual classifier a weight and then utilizes this weight-probabilistic ensemble for further processing. As illustrated in the equation below, the classification is dependent on the measurement of evidence supplied by the individual classifiers. We have

(14) $$class\left(v\right)=arg.max\forall class_{i}\left(\sum_{i}^{c}a_{k}*Pc_{k}\left(y=class_{i}|v\right)\right),$$

where $P_{ck}\left(y=class_{i}|v\right)$ is basically the probability of class i, which gives us the sample node by using the classifier denoted by *k* and the weight is denoted by $a_{k}$ that is linked with the probabilistic prediction of the sample belonging to class $C_{k}$. The data that was utilized for training is 70% and testing was done at 30% of the data comprised of two labels, namely $X_{1}=Like$ and $X_{2}=Dislike$. Figure 7 depicts the proposed ensemble framework for hybrid classifiers. The framework includes an ensemble of feature vectors: $a_{k}=aDNN,aSVM,aDT$. Optimization of the weights in this weight vector was done by using the genetic algorithm. Two steps are involved in the modeling of weights. In the first phase, confused samples are separated from the rest of the samples contained in the training dataset. Confused samples are those samples that are categorized to distinct classes by their respective classifiers. As it is easy and requires less time to process only the confused samples, in the second phase, the weights for the confused samples that were computed in the first phase are optimized. The optimization of weights is carried out by using the genetic algorithm.

Figure 7 shows us the block diagram of ensemble classifier. The genetic algorithm is one of the methods that can be used to eliminate redundant or irrelevant features. In machine learning, often redundant or irrelevant features obscure the primary categorization features. Feature selection has become an important area of study in order to eliminate such characteristics that are unnecessary. The feature-selection process is to select some of the most effective and representative features from a set of features in order to achieve the purpose of reducing the feature space dimension. The genetic algorithm is useful for selecting features. The selection is based on the new individuals’ physical fitness. The genetic algorithm is founded on the idea that the greater the fitness, the greater the probability of selection. With low fitness, the probability of selection is low. This selection technique produces a relatively optimum group from the initial data. The selected individuals then undergo the crossover procedure to produce new individuals. The subsequent step is mutation, which yields a new subset. Through this series of processes, a new generation of individuals is produced that is unique from the original generation and is progressing toward an increase in overall fitness from one generation to the next. As a result of the decision to generate the future generation by picking the individuals that are fit, the less fit individuals would be gradually removed. We have

(15) $$P\left(x_{k}^{i}\right)=\frac{f(x_{k}^{i})}{\sum_{i=1}f{\left(x\right)}_{k}}.$$

Figure 7 shows the weights optimization of classifiers like SVM, DNN and DT.

## 7. Performance Evaluation

To verify the validity of the proposed system, different performance evaluation criteria have been used. These included sensitivity (the true positive rate), specificity (the true negative rate), and ROC (the receiver operating characteristic curve). Accuracy is the measure of correctly classified samples. Accuracy cannot be a good measure of evaluation in our case because even if the system does not correctly identify the positive (which is less in number) but correctly identifies all the instances of the negative class (which has a higher proportion), the accuracy will still be high. Another reason is that with the increase in sensitivity, the false positive rate also starts to increase, measurement of metrics like sensitivity and specificity are required of evaluate our system for classification of samples between the like and dislike states.

- **Accuracy** Classification models can be evaluated by using a variety of criteria, and one of them is accuracy. Accuracy is the percentage of correct predictions out of all possible predictions. The proposed model considers accuracy to be a measure of the model’s ability to accurately predict whether the consumer will like the product or dislike the product. The accuracy can be calculated by using Equation (22):
  (16) $$Accuracy=\frac{TN+TP}{TN+TP+FN+FP}.$$
  In the above equation, *TP* means the true positive and *FN* indicates the false negative.
- **Sensitivity** The percentage of correct positive predictions is what we call sensitivity. Higher sensitivity in a proposed model denotes the model’s ability to accurately predict whether or not the customer will like the product. Equation (23) can be used to calculate sensitivity:
  (17) $$Sensitivity=\frac{TP}{TP+FN}.$$
- **Specificity** The degree of specificity refers to the validity of negative predictions, or the percentage of true predictions. With a better model, we can forecast that a consumer will not like a product with greater accuracy. Equation (24) can be used to calculate sensitivity. We have
  (18) $$Specificity=\frac{TN}{TN+FP},$$
  where *TN* means the true negative and *FP* indicates the false positive. To conclude, the average specificity of the ensemble classifier is better than the average specificity of DT, SVM, and DNN.

## 8. Results and Discussion

In this section, we present the results of our investigation as well as an explanation of the methodology that we have proposed. After the preprocessing and feature extraction, the weights of classifiers like DT, SVM, and DNN were optimized by using the genetic algorithm. Results were compared in terms of the area under curve, accuracy, sensitivity, and specificity, the proposed method is compared with other classifiers, including DT, SVM, and DNN.

An SVM is a type of supervised machine learning model that uses classification techniques in order to categorize samples into one of two groups. After providing an SVM model with training data for each category, it is possible to train the model to categorise fresh data by using the training data. They offer two significant advantages over more recent algorithms such as neural networks: increased efficiency with a lower sample size and faster speed than the older techniques (in the thousands). Because of this, the approach is suitable for text classification tasks, which need a dataset consisting of at least several thousand cases that have been labelled. In the field of machine learning, the measuring of performance is a very important component. The AUC-ROC curve is a useful tool for assessing the performance of classification algorithms. An area under the receiver operating characteristic curve (AUC-ROC) is used to visualise the performance of binary class classifiers.

Of the several approaches to evaluation, the ROC curve is by far the most useful. Sometimes it is also referred to as the area under the receiver operating characteristic (AUROC). The AUC-ROC curve is a useful tool for evaluating the performance of a classification task at a number of different thresholds. When it comes to the ability to separate measurements, the area under the curve (AUC) is the symbol for it, whereas the probability is generated from the ROC curve. When the area under the curve (AUC) is greater than 0.8, it indicates that the model does an excellent job of predicting the proper answer. When plotting the ROC curve, the x-axis indicates the rate of false positives, whereas the y-axis indicates the rate of true positives. As long as the AUC is close to 1, a model is likely to stand out, and when the value of the AUC approaches 1, a model is functioning in a very good way for binary class classification. If the AUC is getting close to 0, it suggests that the model’s output is not very accurate, and is not satisfactory. It suggests that our model is incorrectly forecasting the values 0 and 1, respectively.

**Figure 8:**
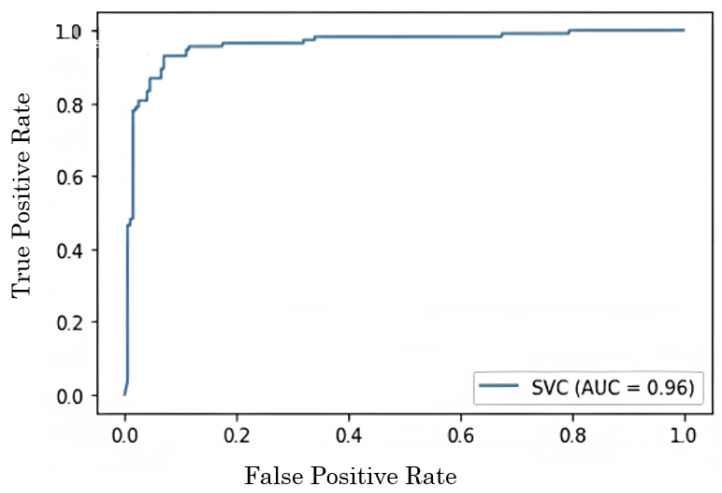
AUC curve to examine the performance of ensemble classifier.

Figure 8 depicts the AUC curve of the classifier. In the proposed methodology, we have compared the accuracy with different classifiers which include decision tree, SVM, DNN, and the proposed hybrid classifier. Among the different classifiers, the hybrid classifier outperforms in terms of accuracy and gives us highest accuracy of approx 96.68%. The sensitivity is also a very important feature to evaluate the performance of the model.

## 9. Result Achieved by Varying Different Experimental Settings

The proposed deep learning model is employed on the mentioned data with the goal of recognition of customer preference in terms of likes and dislikes. The experiment started by preprocessing the data to reduce the data by removing unnecessary information and also noise present in the data. In contrast to previous findings, preference states seem to generate a low-frequency EEG signal. Thus, the useful bandwidth of EEG signal data for preference detection is between 4 and 45 Hz. We have devised three different experiments.The subsequent sections describe the experiment’s goal, the steps that were taken, and the results that were found in each experimental setting.

### 9.1. Analyzing the Effect of Various Preprocessing Techniques in the Proposed System

Increasing the signal-to-noise ratio (SNR) of EEG signals is a key step in the customer preference recognition technique because it lowers noise and other distortions in the signals. EEG signals have been preprocessed by using a variety of approaches to boost SNR and eliminate power line noise. Bandpass filtering, fast Fourier transform (FFT), Savitzky–Golay filter and synthetic data generation are all examples of these approaches. First, the EEG signals were not preprocessed in order to evaluate the performance of the baseline feature-extraction method. By using time/frequency domain characteristics obtained from the data, states of liking and disliking are categorized by using an SVM classifier. Accuracy, sensitivity, and specificity are used to evaluate the experimental environment. Table 3 shows the analysis and comparison of various preprocessing techniques for removal of noise and checks the performance metrics like accuracy, sensitivity, and specificity.

A consumer preference recognition system that does not preprocess EEG signals is unable to obtain improved results. In this experimental setup, we could not achieve a sensitivity and accuracy of more than 70%, with 67.2% sensitivity, and 69.5% specificity. From experiment 1, we concluded that without the preprocessing of EEG signals we could not achieve good results, so in the second experiment a bandstop filter was employed to remove the high frequency components. After applying the bandpass filter and Savitzky–Golay filter and SMOTE technique, an experiment was conducted to check the effect of the preprocessing technique. In the second experiment, it was concluded that by using preprocessing techniques the accuracy improved to 76%.

**Figure 9:**
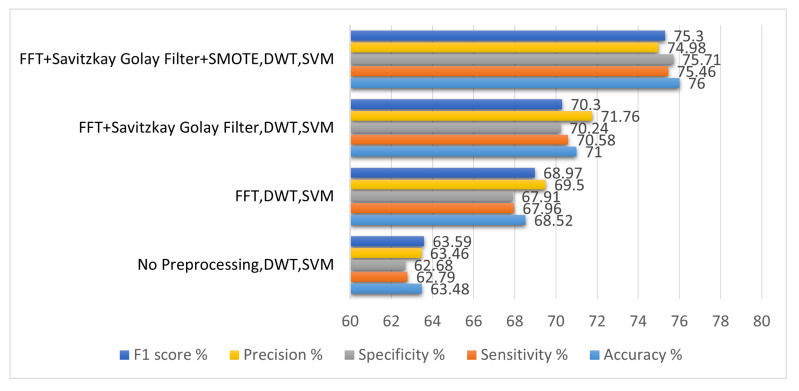
Analysis of different preprocessing techniques in the proposed system.

Figure 9 shows us the comparison of difference preprocessing techniques in terms of specificity, sensitivity, and accuracy.

### 9.2. Examining the Effects of Different Feature-Extraction Methods in the Suggested System

In the second experiment, the optimal method for extracting features from a dataset is examined. The handcrafted features and automated features were tested in this experiment. The handcrafted features include the DWT and power spectral density (PSD), and automated feature include LSTM-based features. Both the handcrafted and automated features were tested one by one, and results were evaluated by accuracy, specificity, and sensitivity. An additional factor is that the automated features were extracted by using LSTM.

In the first iteration, the preprocessing settings were retained the same as they were in the first experiment, and the DWT was utilized in order to extract the features. SVM was employed for classification after feature extraction. This method’s outcomes cannot be compared to what is currently possible. The experiment had a 62.79% sensitivity and a 62.68% specificity. PSD was used instead of DWT in the second iteration, and preprocessing settings were identical to the first experiment. After the feature extraction, classification was done by SVM. As a result of this adjustment, the sensitivity was increased to 78.87%, and the specificity was increased to 78.69%. In the third iteration, DWT- and PSD-based features were concatenated, the preprocessing and classification setting was the same as per a previous experiment. In this iteration, the accuracy achieved was 87.25%, the specificity was 87.78%, and the sensitivity was 87.66%.

Table 4 shows us the comparison of different feature-extraction techniques. In order to better understand the implications of various feature-extraction methods, we designed an LSTM network. The results produced in this environment were somewhat comparable to those of current state-of-the-art systems. After a thorough investigation, similar architectures were also discovered in the literature. The details of this network have been explained in detail in chapter 3. The experimental settings produced the increased accuracy of 85% with specificity of 84.78% and sensitivity of 84.27%. In the last iteration, DWT-, PSD-, and LSTM-based features were gathered, passed to the SVM classifier, and the preprocessing settings were the same as per previous iterations. The accuracy achieved in this experiment was 87.25%, the sensitivity was 87.66%, and the specificity was 87.66%.

**Figure 10:**
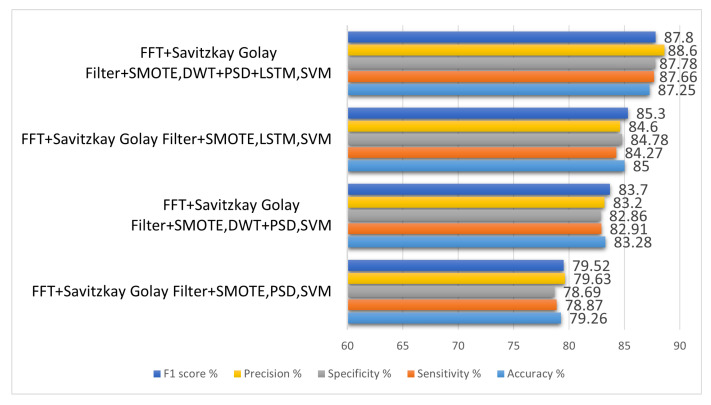
Analysis of different feature extraction techniques in proposed system.

Figure 10 shows us the comparison of feature extraction techniques.

### 9.3. Analysis of Effect of Various Classification Techniques in the Proposed System

Different classifiers were tested as part of a third experimental setting. Preprocessing was handled by Savitzky–Golay filter, SMOTE, and FFT. Feature extraction was handled by DWT and PSD, and LSTM.

DNN was employed to classify in the first iteration. Decision tree was utilised for the classification in the second iteration, and SVM was employed in the third iteration and didn’t get the accuracy. For this reason, weights for all the classifiers are gathered and passed to the genetic algorithm for optimization and then classification between like and dislike was done by optimized weights. The following are the classification results obtained after optimization: a sensitivity of 95.89%, specificity of 96.21%, accuracy of 96.89%, precision of 95.78%, and F1 score of 95.76%. Table 5 summarises the initial conditions and the subsequent outcomes for each iteration.

**Figure 11:**
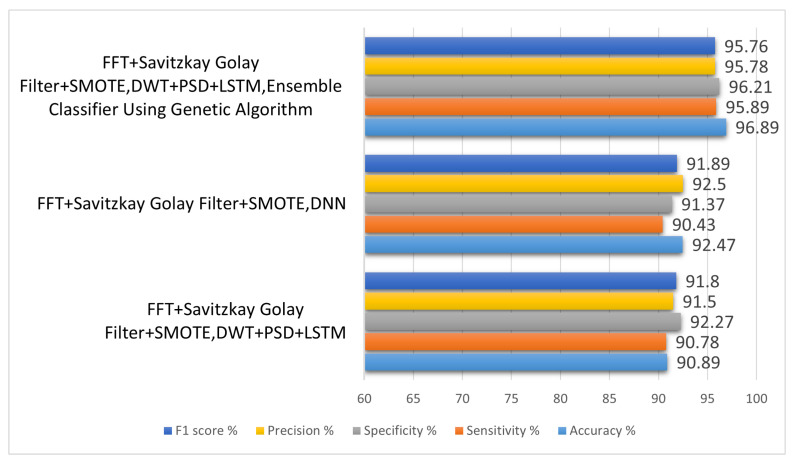
Analysis of different classification techniques in proposed system.

Figure 11 shows us the comparison of different classification techniques.

## 10. Comparison of Results of Consumer Choice Recognition Method by Using Different Experiments

Following the first three experiments, the preprocessing of EEG signals was determined. Figure 4, Figure 5, Figure 6, Figure 7, Figure 8, Figure 9, Figure 10 and Figure 11 compares the results of various experiments conducted as part of this study. In the first experiment, EEG data was used with no preprocessing to extract handcrafted features, which were then classified by using SVM. In the second experiment, EEG signals were preprocessed by first applying bandpass filtering to eliminate high-frequency components; then, handcrafted features were extracted and applied SVM to classify them. The comparison of these two trials reveals that preprocessed signals yield significantly better results with improved accuracy. Following the previous experiment, EEG signals are preprocessed by using a bandpass filter and Savitzky–Golay filter and FFT in the third experiment. In this experiment, the same method was employed for feature extraction and classification as previously used in other experiments. With this experimental setup, there are improvements in accuracy, sensitivity, specificity, precision, and F1 score. Similar to experiment 4, experiment 5, and experiment 6, feature-extraction methods have been modified by adopting the same preprocessing and classification technique. Table 6 depicts the comparison of results for different experimental settings by evaluating in terms of accuracy, sensitivity, specificity, precision, and F1 score.

**Figure 12:**
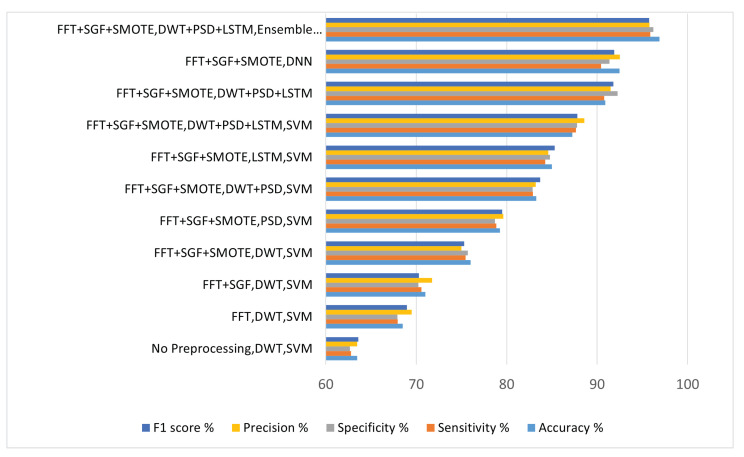
Comparison of results achieved for consumer choice recognition by using different experiments.

Figure 12 shows us the comparison of results achieved for consumer emotion prediction by applying the different experimental settings.

Different experiments were conducted and different kinds of feature-extraction techniques were applied. In first iteration, the features were extracted by using DWT, and experimental settings for preprocessing and classification was the same as previous experiments. By analyzing the results, it was concluded that there is no such improvement in accuracy, specificity, sensitivity, precision, or F1 score. Consequently, concatenation of the DWT and PSD featues was performed, and then the signals were classified in terms of likes and dislikes and came to know that there was improvement in the results. For the next iteration, we have concatenated DWT-, PSD-, and LSTM-based features and compiled the results.The results show that there is a significant amount of improvement in accuracy, specificity, sensitivity, precision, and F1 score by the concatenation of handcrafted features with automated features by LSTM.

In the last experiment, the experimental settings for preprocessing and feature extraction are the same, and classification was iterated by using different classifiers like DNN, SVM, and decision tree. The highest accuracy achieved was 92.68%, which was not acceptable, so the ensemble classifier was employed. The weights for each classifier were passed to the genetic algorithm for optimization. The highest accuracy achieved is 96.89%, with specificity of 96.21%, sensitivity of 95.89%, precision of 95.78%, and F1 score of 95.76%.

**Figure 13:**
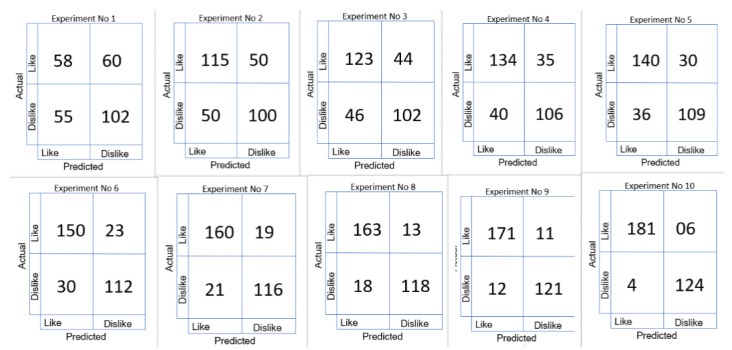
Comparison of confusion matrix for different experimental settings.

Figure 13 shows us the comparison of the confusion matrix for consumer emotion prediction.

## 11. Evaluation of the Effectiveness of the Proposed Method in Comparison to State-of-the-Art Customer Choice Recognition Systems

A dataset consisting of electroencephalogram readings was employed in order to make a comparison between the results obtained by the proposed method and those obtained by traditional consumer choice recognition techniques. The performance of the proposed approach is superior compared to the existing methods.The system for recognizing consumer choice can be adversely affected by an increase in sensitivity with low specificity. The accuracy is 88%, but specificity is 86.76%, which reveals the high false alarm rate that affects the recognition of consumer choice negatively [13]. It is possible to detect the consumer preference on various e-commerce products; however, Jafar et al. [65] were unable to obtain sensitivity and specificity greater than 69%. Aldayel et al. [4] suggested a consumer choice recognition system with an average sensitivity of 91.2%, but only 81.5% specificity. Rupali et al. [57] and Yadava et al. [1] have employed DWT for feature extraction, but the proposed architecture uses automated as well as handcrafted characteristics to classify between likes and dislikes. The proposed method has obtained a sensitivity of 95.89% while maintaining a specificity of 96.21%. The results of the suggested technique are compared with recent state-of-the-art methodologies in Table 7, which examines the three concepts of accuracy, specificity, and sensitivity.

The ROC curve is another crucial efficiency metric. This graph shows how well a classification system performs in terms of sensitivity versus the percentage of false positives. This experimental setting comprises the preprocessing of EEG signals by applying bandpass filtering, FFT, SMOTE, and the Savitzky–Golay filter, as well as the feature extraction and categorization of extensive feature set by using an ensemble classifier. It has been discovered that the method that was proposed is capable of reaching better sensitivity while keeping a low number of false positives. The outcomes of the proposed system are compared with those of the state-of-the-art systems in Table 7. The proposed system has higher levels of both specificity and sensitivity. Therefore, it is crucial to get a high, genuinely positive rate and low false positives by having a class with a high degree of similarity chosen as the positive class. According to the findings, the proposed system outperforms the state of the art in terms of both true positive rates and false positives.

## 12. Conclusions and Future Directions

The first phase in consumer choice recognition often involves the preprocessing of EEG signals, followed by feature extraction and classification. Numerous academics have attempted to forecast customer preferences in terms of likes and dislikes during the past few years. The procedure of gauging customer choice with greater sensitivity and specificity has proven difficult. Effective preprocessing of EEG signals includes removing EEG signals’ noise from EEG signals and to deal with the problem of class imbalance caused by fewer data samples from the like class in comparison with the dislike class and extracting features that give high interclass variance to assist in accurate classification of like and dislike states are some of the issues that must be resolved. Researchers have not been able to enhance classification accuracy without preprocessing of EEG signals; hence, preprocessing and noise removal plays a critical role in attaining improved accuracy. Researchers have adopted the notch, Butterworth, PCA, and ICA for the preprocessing of EEG signals. However, the results reveal that FFT, SMOTE, and bandpass filter do a better job of boosting the SNR than other approaches.

In recent studies researchers used PSD to extract features, whereas decision tree was used for classification. To improve the performance evaluation metrics like accuracy, specificity, and sensitivity, the proposed model was tested by using different classifiers, but the improved accuracy, sensitivity, and specifivity was achieved by an ensemble classifier that classifies the EEG signals by optimizing the weights of classifiers like DNN, SVM, and RF by genetic algorithm. Several gaps were determined in preprocessing and feature extraction after conducting this comparison. Feature extraction implies a customized LSTM architecture. The sensitivity and specificity of these approaches have been assessed. In the existing systems, the problem of class imbalance was not confronted. In the proposed method to deal with class imbalance, the SMOTE technique was employed.

The proposed method makes use of SMOTE to generate like class samples to resolve the problem of class imbalance. For classification of EEG signals, we employed different classifiers but could not achieve the desired accuracy. Consequently, an ensemble classifier was employed, and weights from different classifiers were optimized by using the genetic algorithm. It is significant that our proposed technique has improved sensitivity, specificity, accuracy, precision, and F1 score. In this dataset, three performance indicators have yet to be attained by any existing methods. According to the results, our proposed method’s ROC curve assessment outperforms existing methods in terms of increased sensitivity and specificity. In the future, we can also employ generative adversarial networks (GANs) for synthetic data generation. It is possible that future research will examine different techniques to deal with fake responses. Furthermore, a neutral choice for the products might be implemented to present users with more options. When viewing products, the tracking of a user’s eye movement could be seen as additional parameter in the prediction of preferred choices. To improve the prediction outcomes, it may be necessary to investigate more robust features and classifier combinations. Secondly, for good results we can also focus on the dataset. In this research, the total instances for our ensemble classifier are 1050, which could be considered a small number of examples. Consequently, in the future we can apply our model to the dataset having large instances of customer preferences to obtain better results. 

## Figures and Tables

**Table 1:** Comparison of different state-of-the-art customer choice recognition systems.

Method	Preprocessing	Features	Classifier	Accuracy	Sensitivity	Specificity
Amna et al. [13] (2022)	Savitzkay Golay Filter	-	Boosted Tree Classifier	88.89%	84.68%	86.76%
Abeer al Nafjan et al. [54] (2022)	bandpass Filter	PCA	DNN	94%	-	-
P.Santhiya et al. [55] (2022)	ICA	NW-STFT	SVM	91%	90.23%	89.97%
Somayeh et al. [56] (2022)	ICA	PSD	Statistical Analysis	93%	-	-
Rupali et al. [57] (2021)	bandpass Filter	DWT	LSTM	92%	90.36%	91.86%
Adam et al. [14] (2021)	Notch Filter	PCA	SVM, KNN	68.50%	-	-
Aldayel et. al. [4] (2021)	Bandpass Filter	DWT	DNN	87%	91.2%	87.5%
Yilmaz et al. [58] (2018)	bandpass Filter	Statistical Features	SVM	82.55%	78.63%	80.79%
Jafar et al. [20] (2018)	-	Statistical Features	DT	68.33%	67.98%	66.37%
Yadava et al. [1] (2017)	Savitzky- Golay Filter	DWT	HMM	70%	-	-
Teo et al. [17] (2017)	-	DNN	DNN	74.60%	71.49%	73.60%
Chew et al. [15] (2016)	Average Filter	PSD	SVM	80%	82.3%	80.5%
Maarten et al. [59] (2015)	Notch Filter, ICA	FFT	SVM	68%	-	-
Hakim et al. [52] (2015)	High Pass Filter	Statistical Features	ANN	68.50%	-	-
Ariel et al. [8] (2013)	Low Pass Filter	Statistical Fea tures	Cardinal Analysis	65%	61.73%	64.19%

**Table 2:** Summary of neuromarketing dataset.

**Number of Participants**	25
**Participants Gender/Age**	Both male and females aged 18–38 years
**No. of Products**	14
**No of Samples**	42(14 × 3)
**Total Samples**	42 × 25 = 1050
**EEG Signal Recording Time**	4 s
**No of Classes**	2 (Like & Dislike)
**No of Channels**	14
**EPOC Sampling Frequency**	2048 Hz to 128 Hz
**Device Name**	EMOTIV EPOC
**Device Name**	EMOTIV EPOC
**Experimental Method**	Each user viewed and evaluated his or her preferences for 42 pictures of ecommerce products in form of like or dislike

**Table 3:** Comparison of different preprocessing techniques in terms of accuracy, sensitivity and specificity.

Method	Accuracy%	Sensitivity%	Specificity%	Precision%	F1 Score%
No Preprocessing, DWT, SVM	63.48	62.79	62.68	63.46	63.59
FFT, DWT, SVM	68.52	67.96	67.91	69.5	68.97
FFT + Savitzkay Golay Filter, DWT, SVM	71	70.58	70.24	71.76	70.3
FFT+Savitzkay Golay Filter+SMOTE, DWT, SVM	76	75.46	75.71	74.98	75.3

**Table 4:** Comparison of different feature extraction techniques applied.

Method	Accuracy%	Sensitivity%	Specificity%	Precision%	F1 Score%
FFT + Savitzkay Golay Filter + SMOTE, PSD, SVM	79.26	78.87	78.69	79.63	79.52
FFT + Savitzkay Golay Filter + SMOTE, DWT + PSD, SVM	83.28	82.91	82.86	83.2	83.7
FFT + Savitzkay Golay Filter + SMOTE, LSTM, SVM	85	84.27	84.78	84.6	85.3
FFT + Savitzkay Golay Filter + SMOTE, DWT + PSD + LSTM, SVM	87.25	87.66	87.78	88.6	87.8

**Table 5:** Comparison of different classification techniques in terms of accuracy, sensitivity, specificity, precision, and F1 score.

Method	Accuracy%	Sensitivity%	Specificity%	Precision%	F1 Score%
FFT + Savitzky–Golay Filter + SMOTE, DWT + PSD + LSTM	90.89	90.78	92.27	91.5	91.8
FFT + Savitzkay Golay Filter + SMOTE, DNN	92.47	90.43	91.37	92.5	91.89
FFT + Savitzkay Golay Filter + SMOTE, DWT + PSD + LSTM, Ensemble Classifier Using Genetic Algorithm	96.89	95.89	96.21	95.78	95.76

**Table 6:** Comparison of different experimental settings applied.

Method	Accuracy%	Sensitivity%	Specificity%	Precision%	F1 Score%
No Preprocessing, DWT, SVM	63.48	62.79	62.68	63.46	63.59
FFT, DWT, SVM	68.52	67.96	67.91	69.5	68.97
FFT + SGF, DWT, SVM	71	70.58	70.24	71.	70.3
FFT + SGF + SMOTE, DWT, SVM	76	75.46	75.71	74.98	75.3
FFT + SGF + SMOTE, PSD, SVM	79.26	78.87	78.69	79.63	79.52
FFT + SGF + SMOTE, DWT + PSD, SVM	83.28	82.91	82.86	83.2	83.7
FFT + SGF + SMOTE, LSTM, SVM	85	84.27	84.78	84.6	85.3
FFT + SGF + SMOTE, DWT + PSD + LSTM, SVM	87.25	87.66	87.78	88.6	87.8
FFT + SGF + SMOTE, DWT + PSD + LSTM, DT	90.89	90.78	92.27	91.5	91.8
FFT + SGF + SMOTE, DWT + PSD + LSTM, DNN	92.47	90.43	91.37	92.5	91.89
FFT + SGF + SMOTE, DWT + PSD + LSTM, Ensemble Classifier By Genetic Algorithm	96.89	95.89	96.21	95.78	95.76

**Table 7:** Comparison of different state of the art customer choice recognition systems.

Method	Preprocessing	Features	Classifier	Accuracy	Sensitivity	Specificity
Amna et al. [13] (2022)	Savitzky–Golay Filter	-	Boosted Tree Classifier	88.89%	84.68%	86.76%
Abeer al Nafjan et al. [54] (2022)	bandpass Filter	PCA	DNN	94%	-	-
P.Santhiya et al. [55] (2022)	ICA	NW-STFT	SVM	91%	90.23%	89.97%
Somayeh et al. [56] (2022)	ICA	PSD	Statistical Analysis	93%	-	-
Rupali et al. [57] (2021)	bandpass Filter	DWT	LSTM	92%	90.36%	91.86%
Adam et al. [14] (2021)	Notch Filter	PCA	SVM, KNN	68.50%	-	-
Aldayel et al. [4] (2021)	Bandpass Filter	DWT	DNN	87%	91.2	81.5
Yilmaz et al. [58] (2018)	bandpass Filter	Statistical Features	SVM	82.55%	78.63%	80.79%
Jafar et al [20] (2018)	-	Statistical Features	Decision Tree	68.33%	67.98%	66.37%
Yadava et al [1] (2017)	Savitzky-Golay Filter	DWT	HMM	70%	-	-
Teo et al. [17] (2017)	-	DNN	DNN	74.60%	71.49%	73.60%
Chew et al. [15] (2016)	Average Filter	PSD	SVM	80%	82.3	80.5
Maarten et al. [59] (2015)	-, ICA	FFT	SVM	68%	-	-
Hakim et al. [52] (2015)	-	Statistical Analysis	DT	68.50%	-	-
Ariel et al. [8] (2013)	Low Pass Filter	Statistical Features	Cardinal Analysis	65%	61.73%	64.19%
**Proposed Method**	**Bandpass Filter, Savitzkay Golay Filter, SMOTE**	**LSTM, DWT, PSD**	**Ensemble Classifier**	**96.89%**	**95.89**%	**96.21%**

## Data Availability

Not applicable.
